# Quality of Life of Patients After Treatment for Cancer in the Head and Neck Region: A Case-Control Study

**DOI:** 10.7759/cureus.25800

**Published:** 2022-06-09

**Authors:** Rafael S Caetano, Fernando F Lima, Elâine P Gomes, Luiz E Volpato

**Affiliations:** 1 Medical School, Universidade Federal de Mato Grosso, Cuiabá, BRA; 2 Cuiabá Dental School, Universidade de Cuiabá, Cuiabá, BRA; 3 Department of Dentistry, Hospital de Câncer de Mato Grosso, Cuiabá, BRA

**Keywords:** supportive care needs, oral cavity, radiotherapy, quality of life, head and neck neoplasm

## Abstract

Introduction: It is known that side effects caused by antineoplastic therapy can affect patients' quality of life (QOL). However, the long-term effects on patients’ quality of life are not well known. This study aimed to evaluate patients' quality of life who underwent radiotherapy for head and neck cancer lasting more than six months compared to individuals who did not experience treatment.

Methods: Thirty-three patients who underwent treatment for cancer in the head and neck region for at least six months and sixty-six individuals without cancer matched for age and sex were given the European Organization for Research and Treatment of Cancer QLQ-C30/QLQ-H & N35 questionnaires. Other pertinent information from patients was taken from the hospital chart. The Mann-Whitney nonparametric test was applied to verify the statistical significance of the difference in means between the groups, and a significance of 5% was considered.

Results: Group 1 consisted of 33 patients with a mean age of 63.42 ± 11.25 years; 81.8% were smokers; 84.8% were drinkers; the sites most affected by cancer were the palate/oropharynx and the floor of the mouth (7 cases), and the most common type was epidermoid carcinoma (78.79%). The overall quality of life was 61.62. Among group 2 patients, the average age was 64.27; 84.85% were smokers, and 65.15% were drinkers. The overall quality of life was 71.46 in group 2. There was a group of variables in which the scores of patients without cancer were statistically lower (better quality of life) than those presented by cancer patients, namely, loss of appetite, pain, swallowing, cognitive problems, speech problems, problems eating in public, sexuality, teeth, mouth opening, dry mouth, sticky saliva, nutritional supplements, feeding tube, and weight gain.

Conclusion: Patients who underwent antineoplastic treatment for more than six months had a worse overall quality of life than individuals who did not experience such treatment. These patients had worse results in the components of appetite loss, pain, swallowing, cognitive problems, speech problems, problems with public eating, sexuality, teeth, mouth opening, dry mouth, sticky saliva, nutritional supplement, feeding tube, and weight gain.

## Introduction

The International Agency for Research on Cancer has estimated 354,864 new cases of lip and mouth cancer, 92,887 oropharynx and 52,799 salivary glands worldwide [1]. The treatment of neoplasms in the head and neck region may involve surgery, radiotherapy, and chemotherapy, alone or in combination.

Survival rates of head and neck cancer patients (HNCP) are changing, with a growing number of survivors and a greater length of survivorship; however, these individuals live with short-term toxicities and long-term treatment-related effects [2]. The adverse effects include xerostomia, dysphagia, and trismus, among others [3,4], with a potential impact on a patient’s well-being and quality of life (QOL) [5,6]. These symptoms have been associated with emotional, physical, and social problems that reduce QOL [7,8]. More research is needed to establish the prevalence of treatment-related side effects and their impact on QOL [2].

Thus, this study aims to evaluate the QOL of patients who have completed treatment for head and neck cancer and compare the results found with a group of patients without cancer and treatment.

## Materials and methods

The Research Ethics Committee approved this cross-sectional study of the Universidade de Cuiabá with protocol number 1.852.857.

Ninety-nine patients were divided into two groups: 33 patients who underwent treatment for cancer in the head and neck region (group 1) and 66 individuals without cancer matched for age and sex at a ratio of two to one (group 2). All research subjects signed the informed consent form.

For group 1, the patients were recruited from the Dentistry Department of the Mato Grosso Cancer Hospital, Cuiabá, Brazil. The inclusion criteria were men and women over the age of 18 who underwent treatment for head and neck cancer that was completed at least six months before data collection. It is important to mention that data from patients in this group were previously published in a study that verified the correlation between the three most used instruments for evaluating the QOL of HNCP [9].

Data on age, sex, social habits of smoking and drinking, tumor location and histological type, and oncologic treatment performed by the patients were collected from each patient's medical records.

Patients underwent the European Organization for Research and Treatment of Cancer Quality of Life Questionnaire, Core Module (QLQ-C30), and the Head and Neck Module (QLQ-H&N35).

The questionnaires were administered in the morning when patients had their follow-up appointments at the hospital. The patients were referred to a private room, the questionnaires were self-administered, and only when the patient had any doubts about its completion did the researcher read the question to the patient. For group 2, the patients were enrolled at the Dental Clinic of the Universidade de Cuiabá and the Padre Firmo Community Center in Cuiabá, Brazil. The questionnaire was applied in the same way as in group 1.

The data from the questionnaires were manually transferred to an Excel spreadsheet (Microsoft, Albuquerque, USA) that served as the basis for analysis using the Statistical Package for Social Science (SPSS 20.0) software (IBM, Chicago, USA) and Statistical Analysis System (SAS) 9.0 (StatSoft, Cary, USA).

A descriptive analysis of the patients’ variables was presented using absolute and relative frequencies. Regarding the age of the patients, the mean, standard deviation, and minimum and maximum values ​​found were calculated. The Mann-Whitney test was used to compare the means of the evaluation items between the groups (with and without cancer).

The hypothesis tests in this research considered a significance level of 5%; the null hypothesis was rejected when the p-value was lower than 0.05.

## Results

The total study population consisted of 99 patients, divided into two groups (group 1 with 33 patients and group 2 with 66 patients). The average age presented by group 1 was 63.42 ± 11.25 years, while the average age of group 2 was 64.27 ± 10.62 years.

In group 1, males represented 69.70% of the sample, 81.8% smokers, and 84.8% the drinkers. The most common histological type of tumor was epidermoid carcinoma (78.79%). Of these patients, 63.64% underwent surgery, 90.91% underwent chemotherapy, and 100% underwent radiotherapy (Table 1).

**Table 1:** Distribution of patients according to sociodemographic characteristics, histological type of tumor, and type of treatment.

Variable	Group 1 (n=33)	Group 2 (n=66)
Sex		
Male	23 (69.70%)	42 (63.64%)
Female	10 (30.30%)	24 (36.36%)
Smoker		
Yes	27 (81.8%)	56 (84.85%)
No	6 (18.2%)	10 (15.15%)
Alcoholic		
Yes	28 (84.8%)	43 (65.15%)
No	5 (15.2%)	23 (34.84%)
Tumor histological type		
Metastatic carcinoma	2 (6.06%)	-
Epidermoid carcinoma	26 (78.79%)	-
Mucoepidermoid carcinoma	1 (3.03%)	-
Verrucous carcinoma	2 (6.06%)	-
Adenocarcinoma	2 (6.06%)	-
Surgery		
Yes	21 (63.64%)	-
No	12 (36.36%)	-
Chemotherapy		
Yes	30 (90.91%)	-
No	3 (9.09%)	-
Radiotherapy		
Yes	33 (100%)	-
No	0 (0%)	-

Table 1 also shows data from group 2, where 63.64% were male. Regarding social habits, 84.85% reported being smokers, and 65.15% reported being drinkers.

Through the analysis of the data obtained from the questionnaires, it was observed that the patients from group 1 presented worse Global Quality of Life, with an average equivalent of 61.62 points. In comparison, group 2 presented an average of 71.46 points (Table 2).

**Table 2:** Mean, median, and standard deviation followed by the Mann-Whitney test p-value for the EORTC-QOL C30 and QOL-H&N 35 instruments.

Variables	Group 1 (n=33)			Group 2 (n=66)			p-value
	Mean	Median	Standard deviation	Mean	Median	Standard deviation	
EORTC-QOL C30							
Global Health Status	61.62	50.00	21.34	71.46	66.67	23.80	0.0276*
Physical performance	68.48	66.67	25.40	76.77	80.00	18.90	0.2024
Functional performance	64.65	66.67	34.04	71.72	75.00	28.34	0.3693
Emotional performance	58.08	66.67	30.86	67.17	75.00	25.02	0.2159
Cognitive performance	72.22	83.33	28.77	70.71	75.00	26.96	0.6841
Social performance	89.90	100.00	15.56	90.66	100.00	16.31	0.5815
Fatigue	28.96	22.22	23.56	22.39	22.22	21.92	0.1852
Nausea or vomiting	8.08	0.00	23.24	3.28	0.00	9.35	1.0000
Ache	22.22	16.67	25.23	33.59	33.33	29.88	0.0719
Dyspnea	11.11	0.00	21.52	9.60	0.00	19.18	0.7534
Insomnia	36.36	33.33	40.28	29.29	0.00	35.81	0.4731
Loss of appetite	28.28	0.00	39.19	4.55	0.00	16.42	0.0002*
Constipation	15.15	0.00	27.75	8.08	0.00	18.55	0.2871
Diarrhea	17.17	0.00	26.51	9.60	0.00	25.99	0.0506
Financial difficulties	34.34	33.33	39.52	23.23	16.67	28.63	0.2945
QOL-H&N 35							
Ache	23.23	25.00	18.37	10.23	8.33	11.40	< .0001>
Swallowing	36.62	33.33	23.38	5.18	0.00	12.81	< .0001>
Cognitive problems	36.36	33.33	24.81	5.30	0.00	16.58	< .0001>
Speech problems	18.18	0.00	24.82	4.21	0.00	8.88	0.0029*
Trouble eating in public	33.33	25.00	26.52	5.30	0.00	10.40	< .0001>
Problems with social contact	16.97	6.67	19.44	8.59	6.67	10.70	0.0616
Sexuality	57.07	66.67	37.27	34.85	33.33	34.06	0.0049*
Teeth	59.60	66.67	44.69	36.36	33.33	31.34	0.0092*
Mouth opening	26.26	0.00	39.75	5.05	0.00	13.39	0.0036*
Dry mouth	87.88	100.00	24.75	19.19	0.00	28.08	< .0001>
Sticky saliva	67.68	66.67	35.83	11.11	0.00	18.80	< .0001>
Cough	19.19	0.00	30.08	16.16	0.00	24.97	0.7859
I felt sick	15.15	0.00	27.75	14.14	0.00	24.15	0.8434
Painkillers	45.45	0.00	50.56	53.03	100.00	50.29	0.4822
Nutritional supplements	42.42	0.00	50.19	10.61	0.00	31.03	0.0003*
Feeding tube	45.45	0.00	50.56	0.00	0.00	0.00	< .0001>
Weight loss	42.42	0.00	50.19	31.82	0.00	46.93	0.3026
Weight gain	54.55	100.00	50.56	21.21	0.00	41.19	0.0009*

On the functional scale (physical, performance, cognitive, emotional, and social), the variables showed no significant difference between the groups. Regarding the scale of symptoms or additional problems (fatigue, nausea and vomiting, pain, dyspnea, insomnia, loss of appetite, constipation, diarrhea, and financial difficulties), patients in group 1 had a greater loss of appetite than patients in group 2.

In Table 2, the QOL-H&N 35 questionnaire data show the lowest scores in the items of a problem with social contact and feeling sick. In contrast, the highest scores were for dry mouth sensation and sticky saliva. There was a group of variables in which the scores of the patients in group 1 were statistically lower than those presented by the patients in group 2: loss of appetite, pain, swallowing, cognitive problems, speech problems, problems with public eating, sexuality, teeth, mouth opening, dry mouth, sticky saliva, nutritional supplements, feeding tube, weight gain.

## Discussion

This research showed that even six months after the end of antineoplastic treatment, patients with a history of head and neck cancer still have a lower QOL than individuals of compatible sex and age without a history of cancer. This difference stands out in the global health status and in general items such as loss of appetite and sexuality, but mainly in the structures and functions of the stomatognathic system.

Given the increasing survival of HNCP submitted to antineoplastic therapies, it was suggested that the evaluation of the patients’ QOL be incorporated into clinical practice [9]. Due to the prevalence of long-term treatment-related effects and their impact on patients’ QOL, as seen in this study, we recommend that the evaluation continue periodically after the antineoplastic treatment conclusion.

Several instruments have been proposed to evaluate specifically the QOL of HNCP. The most commonly used tools are the Functional Assessment of Cancer Therapy Quality of Life Measurement System (FACT-H&N), the University of Washington Quality of Life Questionnaire (UW-QOL), and the EORTC QLQ-C30/EORTC QLQ-H&N35 [10]. A previous study found a significant correlation between the three instruments, so regardless of the questionnaire used, the same result in relation to QOL is found either in the overall evaluation of the patient or in the evaluation of the specific domains of pain, appearance, activity, swallowing, chewing, speech, taste, saliva, humor, and anxiety [9]. Thus, the selection of the instrument for research involving this particular kind of patient should consider the specific aspects that one wishes to evaluate. In this study, the EORTC QLQ-C30/EORTC QLQ-H&N35 was the chosen instrument because, unlike the other two questionnaires, its questions and possible answers are more applicable to individuals without head and neck cancer, favoring the comparison between the two patient profiles.

It is worth mentioning that in relation to functional scales and global health status, higher scores in the EORTC QLQ-C30/EORTC QLQ-H&N35 are related to better QOL; however, for the symptom scales, higher scores correspond to a more significant presence of that symptom and, consequently, a worse QOL [11]. That explains why the score of the global health status of the group of individuals who did not undergo antineoplastic treatment had a higher score than the group that underwent treatment, in contrast to the symptom scales where the contrary occurred.

Patients undergoing treatment for head and neck cancer frequently report a loss of appetite [12]. Even after the completion of treatment, loss of appetite continued in this group of patients compared with patients in the control group, proving the importance of continuous nutritional assistance to these patients.

Due to the symptoms of therapy, especially dysphagia, weight loss is a commonly reported side effect. It is also possible to mention the type of diet in this population that often needs a tube to meet nutritional needs [13]. Swallowing, dietary supplements, feeding tube, and weight gain presented were statistically significant, corroborating with other studies [14,15].

The pain was also more present among patients from group 1. It is a symptom frequently associated with cancer and its treatments, and it results in poor QOL due to its influence on function and emotional impact [16], even months after completion of antineoplastic therapy.

A symptom that is not always present in the questionnaires directed to this specific group of patients is sexuality, in which the score of patients from group 2 was less than 2/3 of the score of group 1. This may be related to weight loss, constipation, problems with social contact, appearance, or functional changes such as movement limitation and oral secretions [17].

Specific oral symptoms, such as limitation of mouth opening, sticky saliva, and hyposalivation, contribute to the development or aggravation of oral problems [5,18]. These symptoms are recurrent in HNCP [12,14,16,18,19] and show the importance of periodic routine monitoring of the oral health of these patients.

QOL studies emphasize the importance of recognizing the negative impact of antineoplastic therapies even after their termination by health professionals to minimize their adverse effects on patients [20,21]. This study reinforces the need for long-term follow-up of these patients, as patients may need a long time to recover from the side effects of the disease and its treatment or even live with them permanently.

This study presents the limitations of using a cross-sectional approach in convenience sampling. Longitudinal follow-up of a cohort involving a more sizable number of patients could verify whether the impact on quality of life found in this study would be supported in the long term or if it underwent any change. Thus, we suggest the adoption of this methodological design in future studies.

## Conclusions

Patients who had undergone antineoplastic treatment for more than six months had a worse overall quality of life than individuals who had not experienced such treatment. Patients treated for head and neck cancer had worse results in the following components: appetite loss, pain, swallowing, cognitive problems, speech problems, problems with public eating, sexuality, teeth, mouth opening, dry mouth, sticky saliva, nutritional supplement, feeding tube, and weight gain.
